# Compound Danshen Dripping Pill for Treating Nonproliferative Diabetic Retinopathy: A Meta-Analysis of 13 Randomized Controlled Trials

**DOI:** 10.1155/2017/4848076

**Published:** 2017-08-24

**Authors:** Wenjing Huang, Qi Bao, De Jin, Fengmei Lian

**Affiliations:** ^1^Department of Endocrinology, Guang'anmen Hospital, China Academy of Chinese Medical Sciences, Beixiange 5, Xicheng District, Beijing 100053, China; ^2^China Academy of Chinese Medical Sciences, Beixiange 5, Xicheng District, Beijing 100700, China

## Abstract

**Objective:**

We assess the clinical effect of compound Danshen dripping pill (CDDP) for treating diabetic retinopathy (DR).

**Methods:**

Electronic databases were searched from January 2001 to October 2016 to locate randomized controlled trials (RCTs). Efficacy was measured as main outcome and microaneurysms, hemorrhage, exudate, vision, and fundus fluorescein angiography (FFA) were measured as second outcomes. Methodological quality for each study was evaluated, RevMan 5 software was used to assess treatment effects, and GRADE was used to rate quality of evidence.

**Results:**

We located 13 RCTs and methodological quality was evaluated as high risk. Statistics indicated CDDP for treating DR was better than controls and DR risk was reduced 64% with CDDP (RR: 0.36, *P* = 0.68); retinal microaneurysms (MD = −4.32NO, *P* < 0.00001); retinal hemorrhages (MD = −0.70PD, *P* = 0.03); exudate improvements (MD = −0.09PD, *P* = 0.79); visual changes (MD = −0.12 letter, *P* = 0.006); FFA (RR: 0.40, *P* = 0.003). About GRADE, quality of evidence was “low.”* Conclusion*. CDDP may be safe and efficacious for treating or delaying DR and may improve vision or delay vision loss.

## 1. Introduction

Diabetic retinopathy (DR) is a major microvascular complication of diabetes that can lead to retinal detachment and blindness. Annually, 10–12% of new cases of blindness are attributed to DR and in Holland, as many as 21% of newly diagnosed blindness is due to DR [1–3]. One-quarter of DR patients develop severe visual impairment, attributed to diabetic macular edema and proliferative diabetic retinopathy (PDR) [4]. Once proliferation begins, it causes irreversible visual impairment. Blindness for diabetics in China is 25 times more frequent than in nondiabetics [5, 6] and about 20 million diabetics reside in China now, constituting the second largest diabetes population worldwide [7]. Therefore, preventing DR and reducing diabetic-induced visual impairment are key concerns.

DR treatment includes systemic therapy to control glucose, blood pressure, and serum lipids as well as ocular drugs. Severe nonproliferative diabetic retinopathy (NPDR) and PDR have been treated with laser therapy, which prevented vision impairment and loss but did not improve visual acuity. Also, laser treatment may reduce visual field. For NPDR, drug intervention can delay progression of DR, improve visual function, and reduce side effects of laser treatment. Thus, the ability to predict progression in early stages of DR and drug intervention are important for reducing the risk of DR. At this time, no effective treatment for DR exists and nothing has been shown to slow or reverse visual impairment.

The pathogenesis of DR is thought to be poor circulation which damages collateral eye vessels [8], so improved circulation is a therapeutic strategy. The herb* Salvia miltiorrhiza* (Danshen dripping pill, CDDP) was tested in American FDAII clinical trials for safety and efficacy for treating cardiovascular conditions such as myocardial infarction [9]. Also, CDDP is being studied to treat DR [10], although the data quality was poor and safety was not confirmed. Thus, we performed a meta-analysis of randomized clinical trials (RCTs) to compare CDDP and placebo or approved therapies to assess any curative effect and safety of this drug.

## 2. Methods

### 2.1. Data Sources and Search Strategy

We searched the Chinese National Knowledge Infrastructure (CNKI), Chinese Scientific Journal Database (VIP), Chinese Biomedical Literature Database (CBM), WanFang Databases, PubMed, Medline, and Cochrane Library. We used these keywords: (“diabetic retinopathy” OR “diabetic eye diseases”) AND (“compound danshen dripping pill” OR “a danshen-containing Chinese herbal medicine”) AND (“randomized controlled trial” OR “controlled clinical trial” OR “random” OR “randomly” OR “randomized” OR “control”). We searched all articles published from January 2001 to October 2016.

### 2.2. Selection of Studies

Randomized controlled trials (RCTs) were selected which included CDDP as the main intervention and this was compared to approved therapy or controls. The primary outcome was efficacy and secondary outcomes were microaneurysms, hemorrhage, exudate, vision, and FFA. Study participants were diagnosed with DR. Medical record reviews, retrospective studies, repeated reports of research studies, and animal experiments were excluded.

### 2.3. Data Extraction and Quality Assessment

Two authors independently extracted data (W. J. Huang and F. M. Lian) such as author, year of publication and country, sample size, age, sex, intervention (components of intervention), intervention details, dose, treatment duration, changes in microaneurysms, hemorrhage, exudate, visual acuity, and FFA. For incomplete and suspicious data, authors were contacted by e-mail or phoned to obtain the information, but no author offered information. Disagreements over study eligibility were resolved with discussion with a third reviewer (Q. Bao). The Cochrane Handbook for Systematic Review was used to assess study quality [24]. Bias assessment criteria included adequate sequence generation, allocation concealment, blinding of participants and personnel, blinding of outcome assessment, incomplete outcome data addressed, being free of selective reporting, and being free of other biases. We judged each item using three levels (“Yes” means low risk of bias, “No” means high risk of bias, and “Unclear” means other biases); see Table 2.

### 2.4. Data Analyses

Revman 5.0 software was used to analyze data, which was provided by the Cochrane Collaboration. The risk ratio (RR) for data was used as pooled statistics, and weighted mean difference (WMD) of measurement data was used as pooled statistics, and 95% confidence intervals (CI) were calculated [25]. Heterogeneity was assessed by WHAT test and *I*^2^ > 50% or *P* < 0.1 was used to assess significance, and a random effects model was used to explain possible causes of heterogeneity. If *I*^2^ < 50%, there was no heterogeneity and a fixed effect model was used [26]. Publication bias was assessed using a funnel plot [27].

## 3. Results

### 3.1. Study Characteristics

**Figure 1:**
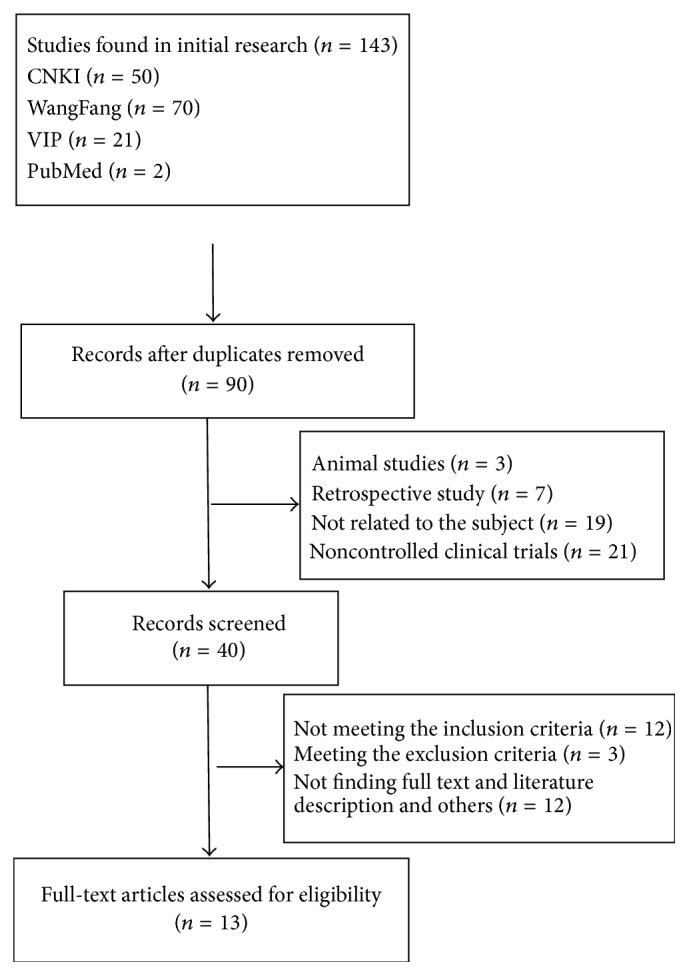
Flow chart of studies selection process.

143 studies were initially identified; finally, 13 studies [11–22] remained after eliminating duplications, examining titles and abstracts, and reviewing full texts.  Figure 1 depicts trial selection and number of studies found. The 13 studies included 874 participants, 477 males and 397 females. Of these, 448 were participants in the intervention group, and 426 were controls. All participants were inpatients/outpatients of the Endocrine Department and they were aged 30 to 80 years. Diagnostic criteria of the 13 studies were based on the accepted and authoritative diagnostic criteria for DR [28–31]. In intervention group, 10 studies [11, 13–15, 17–20, 22, 23] used CDDP alone, and 3 studies [12, 16, 21] used CDDP plus conventional drugs. In control group, 2 studies [20, 21] used placebo, 4 studies [11, 13–15] used vitamin B1 and/or LuDing tablets and/or PanShengDing tablets and/or inosine tablets, and 7 studies [16–22] used calcium dobesilate. The follow-up period ranged from 2 to 6 months, and selected studies are summarized in Table 1.

### 3.2. Quality Assessment

Quality assessments are summarized in Table 2. Only 1 study described methods adequately [23]. The table addresses the remaining study deficits. No study clearly described method of allocation concealment and blinding procedures. All studies showed the baseline data of the two groups were “comparable,” and quit and lost to follow-up cases and adverse events were not recorded. The methodological quality was assessed to be of high risk.

### 3.3. Effect of the Interventions

#### 3.3.1. Efficacy

**Figure 2:**
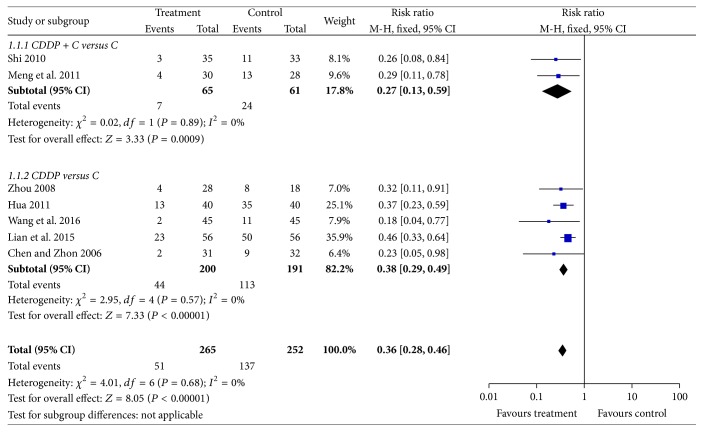
Comparison of efficacy of CDDP.

According to* Guiding Principles of Clinical Research on Chinese Medicine Traditional and New Drugs, *efficacy was assessed based on fundal improvements measured with ophthalmoscopy, fundus fluorescein angiography (FFA) to assess microaneurysms, hemorrhage and exudate areas, and improved vision using a visual chart [28]. Efficacy was evaluated being significantly effective, effective, and ineffective. Total efficacy was evaluated based on significantly effective and effective data, and this was a chief overall outcome. Seven studies were examined [13, 16–19, 21, 23] and their data were homogenous and DR risk was reduced 64% by CDDP (RR: 0.36, Chi^2^ = 4.01, *P* = 0.68, *I*^2^ = 0%, 95% CI [0.28 to 0.46], and *P* < 0.00001). Thus, a fixed effects model was used for statistical analysis and data showed that treatment groups fared significantly better than controls. To compare curative effects of treatment and controls, subgroup was used. Two studies [16, 21] used CDDP plus conventional drugs for treatment, and five studies [13, 17–19, 23] only used CDDP for treatment. Two kinds of studies suggested that treatment was better than controls (*N* = 126, RR: 0.27, 95% CI [0.13 to 0.59], *Z* = 3.33, and *P* = 0.0009 and *N* = 391, RR: 0.38, 95% CI [0.29 to 0.49], *Z* = 7.33, and *P* < 0.00001) and these data appear in Figure 2.

#### 3.3.2. Microaneurysms

**Figure 3:**
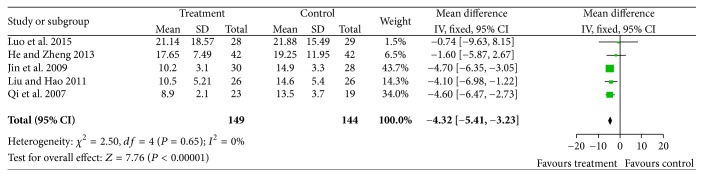
A comparison of the effectiveness of CDDP on retinal microaneurysms improvement.

Five studies [11, 12, 15, 20, 22] reported participants who had more microaneurysms (Chi^2^ = 2.50, *P* = 0.65, and *I*^2^ = 0%). Thus, fixed effects model was used for statistical analysis. Microaneurysms were significantly improved in the treatment group compared with controls (*N* = 293, *Z* = 7.76, MD = −4.32, 95% CI [−5.41 to −3.23], and *P* < 0.00001); see Figure 3.

#### 3.3.3. Hemorrhages

**Figure 4:**
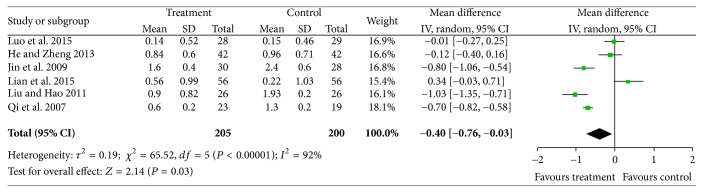
A comparison of the effectiveness of CDDP on hemorrhage improvement.

Six studies [11, 12, 15, 20, 22, 23] provided data for improvements of retinal hemorrhages. Significant heterogeneity was found among these six studies (Chi^2^ = 65.52, *P* < 0.00001, and *I*^2^ = 92%). Using a random effects model, we confirmed a significant difference between treatments and controls (*N* = 405, *Z* = 2.14, MD = −0.70, 95% CI [−0.76 to −0.03], and *P* = 0.03); see Figure 4.

#### 3.3.4. Exudate

**Figure 5:**
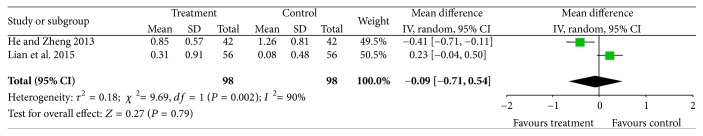
A comparison of the effectiveness of CDDP on exudate improvement.

Two trials [12, 23] provided data for exudate, and they did not show homogeneity (Chi^2^ = 9.69, *P* = 0.002, and *I*^2^ = 90%). A random effects model indicated that there were no significant differences between treatment groups and controls so there may have been differences in intervention measures, observation methods, or periods of intervention (*N* = 196, *Z* = 0.27, MD = −0.09, 95% CI [−0.71 to 0.54], *Z* = 0.27, and *P* = 0.79); see Figure 5.

#### 3.3.5. Visual

**Figure 6:**
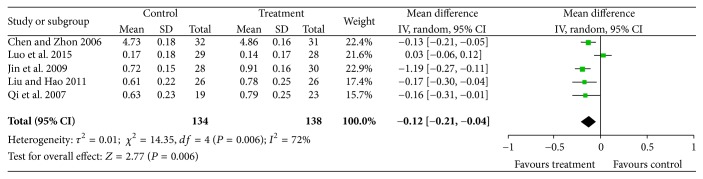
CDDP efficacy for vision improvement.

Five studies [11, 15, 17, 20, 22] reported data for visual acuity and significant heterogeneity was found (Chi^2^ = 14.35, *P* = 0.006, and *I*^2^ = 72%). A random effects model indicated that there were significant differences between treatment groups and controls (*N* = 272, *Z* = 2.77, MD = −0.12, 95% CI [−0.21 to −0.07], *Z* = 2.77, and *P* = 0.006); see Figure 6.

#### 3.3.6. Fundus Fluorescein Angiography (FFA)

**Figure 7:**
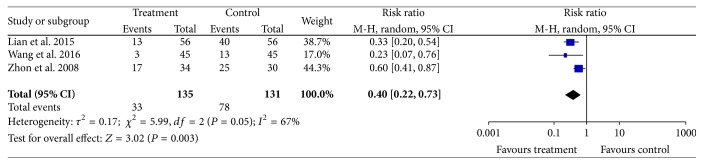
CDDP efficacy for FFA.

Three studies [18, 19, 23] provided data for fundal improvements and they did not show homogeneity (Chi^2^ = 5.99, *P* = 0.05, and *I*^2^ = 67%). A random effects model indicated that treatment groups improved more than controls (*N* = 266, *Z* = 3.02, RR: 0.40, 95% CI [0.22 to 0.73], and *P* = 0.003); see Figure 7.

### 3.4. Publication Bias

**Figure 8:**
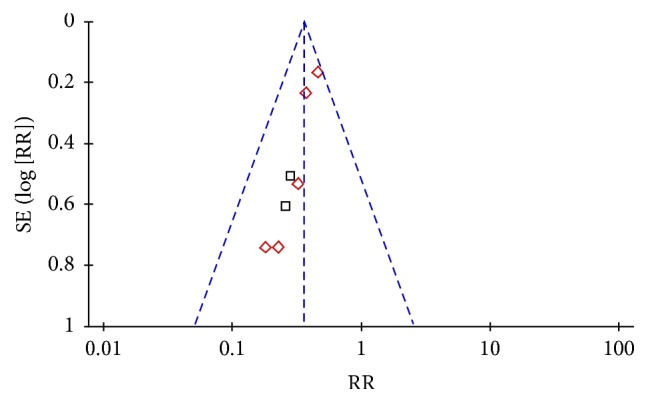
Funnel plot of publication bias.

An “inverted funnel” pattern analysis was used to confirm publication bias and the asymmetrical figure indicated potential publication bias that might influence results (Figure 8). Although we conducted comprehensive search to avoid bias, some negative results may not have been published. Study quality was not homogeneous, and most studies did not indicate randomization approaches. Other deficits may have compromised bias. Even so, included studies had definite diagnostic criteria, and baselines of treatment groups and controls were comparable. Thus, we conclude that CDDP improve fundal lesions of DR participants.

### 3.5. Result of GRADE

GRADE provides a clear and comprehensive methodology for rating the confidence in estimates (quality of evidence). It was used to evaluate effects of CDDP on DR and we confirmed a low quality of evidence. Better high-quality RCTs are needed to confirm the effect of CDDP, Table 3.

### 3.6. Adverse Events

No adverse events were recorded in any study, suggesting that CDDP may be safe.

## 4. Discussion

Our data suggest that 270–540 mg CDDP may be used to treat DR as improvements in various indicators were noted. Of the 13 studies reviewed, the curative effect of CDDP for DR was shown to be superior to controls and this was significantly different for vision improvements. This suggests that CDDP may delay vision loss and retard the progression of DR. The advantages of studies included definite diagnostic criteria and “comparability” for baseline data. But studies were of high risk methodological quality and samples were small. There was no multicenter trial or large samples for collaborative research. Only one study described the randomization method. Many participants were lost and not followed-up! There are five proposals: first, record in detail data of participants who were lost to follow-up or quit midway; second, record long follow-up and record important clinical outcomes after treatment, such as progression of retinopathy to PDR or sustained visual loss; third, methodological quality of clinical studies should use allocation concealment and complete outcome data should be addressed to prevent bias [32]; fourth, use the internationally accepted, uniform, and objective indicators of curative effect [33, 34]; fifth, for search, the negative result of studies must be included.

CDDP is compounded from extracts of* S. miltiorrhiza*, notoginseng* (Panax notoginseng)* and borneol. These three traditional Chinese medicines are widely used and have a long history of treatment in China.* S. Miltiorrhiza *and* Panax notoginseng* are used to treat cardiovascular conditions [35, 36].* S. miltiorrhiza* contains a water soluble tanshinol which allegedly can decrease coagulation, increase fibrinolytic activity, inhibit thrombosis, platelet synthesis, and prostacyclin release, as well as block hydroxyl radical production, prevent lipid peroxidation, and scavenge free radicals [37].

Mechanisms underlying CDDP may include free radical scavenging as* S*.* miltiorrhiza* ketone IIA can inhibit lipid peroxidation and reduce free radicals to protect endothelial diastolic function and vision and visual acuity was reportedly improved after treatment with CDDP [38, 39]. Improved microvascular structure was noted after CDDP treatment. The average thickness of micrangium decreased and diameters widened after CDDP for intake 3 months [18]. CDDP may improve microcirculation near the retina, opening capillaries, and relieving tissue ischemia and hypoxia and this can reduce DR symptoms or delay disease progression [40]. Endothelial dysfunction is prominent in hypercholesterolemic patients and it may contribute to DR by endothelial dysfunction [41]. CDDP may improve lipid metabolism. Hyperlipidemia damages the vascular wall and causes endothelial dysfunction, changing cell membrane structure and leading to microthrombi and ultimately causing DR. CDDP may then delay progression of DR by reducing blood lipids and improving blood flow [42, 43]. CDDP uses multiple sites, multiple pathways, and multitargets. It is characterized by convenient taking, rapid onset of action, and no obvious toxicity or adverse reactions.

## 5. Conclusion

From this meta-analysis, we find that CDDP can be safe and efficacious retarding the progression of DR and delaying vision loss; thus it may be considered as an alternative way to treat DR. But the methodological quality was assessed to be of high risk and GRADE quality of evidence was “low.” Thereby, large-sample, high-quality randomized controlled clinical trials are warranted in the future.

## Figures and Tables

**Table 1:** Characteristics of included RCTs.

Trials	Sample (*N*)	Male : female	Age (years)	Experimental	Control	Duration (months)	Outcomes measured
Qi et al. 2007 [11]	42 (23/19)	25 : 17	36–72	CDDP	Vitamin B1 + LuDing tablets	3	Visual acuity, hemorrhage, microaneurysm
He and Zheng 2013 [12]	84 (42/42)	48 : 36	32–70	CDDP	Placebo	2	Visual, exudate, hemorrhage, microaneurysm
Xu 2011 [13]	80 (40/40)	39 : 41	52.3 54.5	CDDP	LuDing tablets + vitamin C + PanShengDing tablets	3	Efficacy, microaneurysm
Zhon et al. 2008 [14]	64 (34/30)	38 : 26	32–72 34–74	CDDP	LuDing tablets + vitamin C	3	Efficacy, FFA
Liu and Hao 2011 [15]	52 (26/26)	30 : 22	39–76	CDDP	LuDing tablets + vitamin C + inosine tablets	3	Visual acuity, hemorrhage, microaneurysm
Meng et al. 2011 [16]	58 (30/28)	39 : 19	50.60 ± 8.70 51.20 ± 7.90	CDDP + calcium dobesilate	Calcium dobesilate	6	Efficacy
Chen and Zhon 2006 [17]	63 (31/32)	32 : 31	54.60 ± 10.40 58.12 ± 9.31	CDDP	Calcium dobesilate	3	Efficacy, visual acuity
Zhou 2008 [18]	46 (28/18)	23:23	50.40 ± 8.70 50.50 ± 9.36	CDDP	Calcium dobesilate	6	Efficacy
Wang et al. 2016 [19]	90 (45/45)	47 : 43	47–77 48–76	CDDP	Calcium dobesilate	2	Efficacy, visual acuity, FFA
Jin et al. 2009 [20]	58 (30/28)	31 : 27	62.78 ± 7.69 61.11 ± 7.27	CDDP	Calcium dobesilate	3	Visual acuity, hemorrhage, microaneurysm
Shi 2010 [21]	68 (35/33)	36 : 32	38–76	CDDP + calcium dobesilate	Calcium dobesilate	3	Efficacy
Luo et al. 2015 [22]	57 (28/29)	37 : 20	59.54 ± 7.46 57.8 ± 10.03	CDDP	Calcium dobesilate	3	Visual acuity, hemorrhage, microaneurysm
Lian et al. 2015 [23]	112 (56/56)	52 : 60	58.97 ± 7.6 58.97 ± 8.1	CDDP	Placebo	6	Exudate, hemorrhage, FFA, microaneurysm

**Table 2:** Quality assessment of enrolled RCTs.

Studies	Adequate sequence generation	Allocation concealment	Blinding of participants and personnel	Blinding of outcome assessment	Incomplete outcome data addressed	Free of selective reporting	Free of other biases	Risk of bias
Qi et al. 2007 [11]	Unclear	Unclear	No	Unclear	Yes	No	Unclear	High
He and Zheng 2013 [12]	Unclear	Unclear	Unclear	Unclear	Yes	No	Unclear	High
Xu 2011 [13]	Unclear	Unclear	Unclear	Unclear	Yes	No	Unclear	High
Zhon et al. 2008 [14]	Unclear	Unclear	Unclear	Unclear	No	Yes	Unclear	High
Liu and Hao 2011 [15]	Unclear	Unclear	Unclear	Unclear	Yes	No	Unclear	High
Meng et al. 2011 [16]	Unclear	Unclear	Unclear	Unclear	No	Yes	Unclear	High
Chen and Zhon 2006 [17]	Unclear	Unclear	Unclear	Unclear	No	Yes	Unclear	High
Zhou 2008 [18]	Unclear	Unclear	Unclear	Unclear	Yes	No	Unclear	High
Wang et al. 2016 [19]	Unclear	Unclear	Unclear	Unclear	Yes	No	Unclear	High
Jin et al. 2009 [20]	Unclear	Unclear	Unclear	Unclear	Yes	No	Unclear	High
Shi 2010 [21]	Unclear	Unclear	No	Unclear	Yes	No	Unclear	High
Luo et al. 2015 [22]	Unclear	Unclear	Unclear	Unclear	Yes	No	Unclear	High
Lian et al. 2015 [23]	Yes	Yes	Yes	Yes	Yes	Yes	Yes	Low

**Table 3:** Result of GRADE.

Quality assessment							Summary of findings				
Participants (studies) follow-up	Risk of bias	Inconsistency	Indirectness	Imprecision	Publication bias	Overall quality of evidence	Study event rates (%)		Relative effect (95% CI)	Anticipated absolute effects Time frame is 2 months to 6 months	
							With control group	With treatment group		Risk with control group	Risk difference with treatment group (95% CI)
*Efficacy* (critical outcome)											
517 (7 studies) 2–6 months	Serious^1^	No serious inconsistency	No serious indirectness	No serious imprecision	Reporting bias strongly suspected^2^	⊕⊕⊝⊝ *LOW*^1,2^ due to risk of bias, publication bias	115/252 (45.6%)	214/265 (80.8%)	*RR 0.36* (0.28 to 0.46)	*Study population*	
										*456 per 1000*	*292 fewer per 1000* (from 246 fewer to 329 fewer)
										*Moderate*	
										—	

*Microaneurysms* (better indicated by lower values)											
293 (5 studies) 2-3 months	Serious^1^	No serious inconsistency	No serious indirectness	No serious imprecision	Reporting bias strongly suspected^2^	⊕⊕⊝⊝ *LOW*^1,2^ due to risk of bias, publication bias	144	149	—		The mean microaneurysms in the intervention groups were *4.32 lower* (5.41 to 3.23 lower)

*Hemorrhage* (better indicated by lower values)											
405 (6 studies) 2–6 months	Serious^1^	Serious^3^	No serious indirectness	No serious imprecision	Reporting bias strongly suspected^2^	⊕⊕⊝⊝ *LOW*^1,2,3^ due to risk of bias, inconsistency, publication bias	200	205	—		The mean hemorrhage in the intervention groups was *0.40 lower* (0.76 to 0.03 lower)

*Exudates* (better indicated by lower values)											
196 (2 studies) 2–6 months	Serious^1^	Serious^3^	No serious indirectness	No serious imprecision	Reporting bias strongly suspected^2^	⊕⊕⊝⊝ *LOW*^1,2,3^ due to risk of bias, inconsistency, publication bias	98	98	—		The mean exudates in the intervention groups were *0.09 lower* (0.71 lower to 0.54 higher)

*Vision* (better indicated by lower values)											
272 (5 studies) 3 months	Serious^1^	No serious inconsistency	No serious indirectness	No serious imprecision	Reporting bias strongly suspected^2^	⊕⊕⊝⊝ *LOW*^1,2^ due to risk of bias, publication bias	134	138	—		The mean vision in the intervention groups was *0.12 lower* (0.21 to 0.04 lower)

*FFA*											
266 (3 studies) 2–6 months	Serious^1^	No serious inconsistency	No serious indirectness	No serious imprecision	Reporting bias strongly suspected^2^	⊕⊕⊝⊝ *LOW*^1,2^ due to risk of bias, publication bias	78/131 (59.5%)	33/135 (24.4%)	*RR 0.4* (0.22 to 0.73)	*Study population*	
										*595 per 1000*	*357 fewer per 1000* (from 161 fewer to 464 fewer)
										*Moderate*	
										—	

^1^Assessed risk of bias according to six items:adequate sequence generation, allocation concealment, blinding of participants and personnel, blinding of outcome assessment, incomplete outcome data addressed, and being free of selective reporting; we assessed high risk according to quality assessment criteria; ^2^check the publication bias of the systematic review that used the method of “inverted funnel” pattern analysis; the figure was asymmetrical, which showed that potential publication bias might influence the results of this paper; ^3^95% confidence intervals of 14 studies overlap are poor.
